# Identification of microRNAs dysregulated in cellular senescence driven by endogenous genotoxic stress

**DOI:** 10.18632/aging.100571

**Published:** 2013-06-28

**Authors:** Lolita S. Nidadavolu, Laura J. Niedernhofer, Saleem A. Khan

**Affiliations:** ^1^ Department of Microbiology and Molecular Genetics, University of Pittsburgh School of Medicine, Pittsburgh, PA 15219, USA; ^2^ Department of Metabolism and Aging, Scripps Florida, Jupiter, FL 33458, USA

**Keywords:** MicroRNA, aging, cellular senescence, DNA repair, mouse embryonic fibroblasts

## Abstract

XFE progeroid syndrome, a disease of accelerated aging caused by deficiency in the DNA repair endonuclease XPF-ERCC1, is modeled by *Ercc1* knockout and hypomorphic mice. Tissues and primary cells from these mice senesce prematurely, offering a unique opportunity to identify factors that regulate senescence and aging. We compared microRNA (miRNA) expression in Ercc1^−/−^ primary mouse embryonic fibroblasts (MEFs) and wild-type (WT) MEFs in different growth conditions to identify miRNAs that drive cellular senescence. Microarray analysis showed three differentially expressed miRNAs in passage 7 (P7) Ercc1^−/−^ MEFs grown at 20% O_2_ compared to Ercc1^−/−^ MEFs grown at 3% O_2_. Thirty-six differentially expressed miRNAs were identified in Ercc1^−/−^ MEFs at P7 compared to early passage (P3) in 3% O_2_. Eight of these miRNAs (miR-449a, miR-455*, miR-128, miR-497, miR-543, miR-450b-3p, miR-872 and miR-10b) were similarly downregulated in the liver of progeroid Ercc1^−/Δ^ and old WT mice compared to adult WT mice, a tissue that senesces with aging. Three miRNAs (miR-449a, miR-455* and miR-128) were also downregulated in Ercc1^−/Δ^ and WT old mice kidneys compared to young WT mice. We also discovered that the miRNA expression regulator Dicer is significantly downregulated in tissues of old mice and late passage cells compared to young controls. Collectively these results support the conclusion that the miRNAs identified may play an important role in staving off cellular senescence and their altered expression could be indicative of aging.

## INTRODUCTION

MicroRNAs (miRNAs) are ~22 nucleotide long, single-stranded, non-coding RNAs that regulate gene expression. They generally bind the 3' untranslated regions (UTRs) of target mRNAs with varying affinity, resulting in mRNA degradation or inhibition of protein translation [1]. The biogenesis of miRNAs is well-characterized, although the mechanisms by which they participate in post-transcriptional silencing are still being elucidated [2, 3]. A wide range of disease processes including cancer are regulated by miRNAs [4].

MicroRNAs are implicated in the regulation of cellular senescence and aging. Cellular senescence, a state of permanent cell cycle arrest, is hypothesized to be a double-edged sword by suppressing cancer progression while promoting growth inhibition and aging-related tissue degeneration [5]. Cellular senescence can result from events such as telomere shortening, activation of DNA damage response (DDR), epigenetic stresses, or expression of oncogenes such as Ras [6-9]. DDR signaling can also occur in senescent cells with minimal DNA damage, termed the “pseudo-DNA damage response” [10]. This phenomenon occurs in cells ectopically expressing p16 and p21 as well as in cells treated with epigenetic modifiers, such as the histone deacetylase inhibitor sodium butyrate [10, 11]. Senescent cells exhibit a unique gene expression profile, termed senescence-associated secretory phenotype (SASP), and promote the release of pro-inflammatory markers, including IL-6 [12]. The P16INK4A (p16) tumor suppressor is significantly upregulated in senescent cells [13]. A recent paper describing the selective apoptosis of p16-expressing cells in a progeroid mouse (BubR1) directly connects accumulation of senescent cells and age-associated phenotypes in mice [14].

Senescent fibroblasts displaying high SASP express miR-146a/b, which attenuates SASP activity in a negative-feedback loop [15]. MiR-22, miR-519, miR-152 and miR-181a, among others, were recently identified as inducers of cellular senescence [16-18]. Knocking down Dicer, a key component in the miRNA processing pathway, causes inhibition of miRNA biogenesis and results in cellular senescence via activation of p53 signaling [19]. Also, MEF immortalization results in the downregulation of certain tumor suppressor miRNAs such as miR-21, miR-28 and miR-34a [20]. These data suggest that miRNAs are critical for regulating senescence.

The insulin-like growth factor (IGF) signaling pathway is involved in regulating lifespan and mouse mutants with reduced IGF signaling have lifespans 40-70% greater than those of wild-type (WT) mice [21-23]. Several studies examining miRNA dysregulation in aging have utilized naturally aged or long-lived animals. MicroRNA profiling studies examined liver, brain, plasma and peripheral blood mononuclear cells from normally aged mice as well as liver from long-lived Ames Dwarf mouse [24-27]. Additional studies have shown that the neuroprotective effects observed in calorie restricted mice is partly due to downregulation of miRNAs that target the pro-survival gene Bcl-2 [28]. Human centenarian studies have also demonstrated miRNA expression differences between blood samples from long-lived humans and young controls [29, 30]. MicroRNAs are also useful biomarkers in early and non-invasive detection of mild cognitive impairment [31]. However, there are few studies that link both senescence and aging with changes in miRNA expression.

To address this gap in knowledge, we measured miRNA expression in a murine model of a progeroid syndrome in tissues where senescence is established, as well as tissues of naturally aged mice and senescent primary mouse embryonic fibroblasts. The XFE progeroid syndrome is a disease of accelerated aging caused by a deficiency in the XPF-ERCC1 DNA repair endonuclease. Murine models of XFE progeroid syndrome (*Ercc1*^−/−^ knock-out and *Ercc1*^−/Δ^ hypomorphic mice) are well-characterized models that mimic the histopathology of normal aging [32-36]. XPF-ERCC1 is essential for nucleotide excision repair (NER) of helix-distorting monoadducts, repair of DNA interstrand crosslinks, as well as repair of some double-strand breaks [37-39]. *Ercc1*^−/^^Δ^ mice accumulate oxidative DNA damage more rapidly than WT mice, and this is presumed to drive their accelerated aging [40]. Liver transcriptome analysis of *Ercc1*^−/−^ and *Ercc1*^−/^^Δ^ mice revealed gene expression profiles similar to those of livers from aged WT mice, including a decrease in the IGF-1/somatotrophic axis and carbohydrate metabolism [32]. Additionally, *Ercc1*^−/^^Δ^ mice also demonstrate increased p16 expression, cellular senescence, and nuclear abnormalities that are similar to those observed in WT old mice [35].

Primary mouse embryonic fibroblasts (MEFs) undergo stress-related senescence due to growth conditions, in particular the supra-physiological concentrations of oxygen in standard tissue culture conditions [41]. When MEFs are grown in 3% O_2_ conditions, they show no signs of cellular senescence and behave similarly to human fibroblasts expressing telomerase [41]. MEFs grown in 20% O_2_ have three-fold more DNA damage than human fibroblasts grown at 20% O_2_, and have more chromosomal breaks than MEFs grown in 3% O_2_, further underscoring the exquisite sensitivity of MEFs to oxidative damage [41]. *Ercc1*^−/−^ MEFs, which are deficient in DNA repair, quickly accumulate global DNA damage and demonstrate cellular senescence phenotypes at earlier passages compared to WT MEFs [32, 42]

An additional benefit of using progeroid mouse models to identify dysregulated miRNAs in tissues is that studies can be performed in a relatively short period of time compared to studies using normal or long-lived animal models. A previously published study demonstrated the usefulness of progeroid models, in particular the Zmpste24-null mouse modeling Hutchinson-Guilford Progeria Syndrome, in identifying miRNAs regulating organismal aging. This study showed that the miR-29 family is linked to the cellular DNA damage response and when upregulated in a p53-dependent manner, miR-29b reduces cell proliferation and increases cellular senescence [43]. Mir-29 acts as a tumor-suppressive, pro-aging molecule via chronic activation of p53 signaling [43].

We wished to identify miRNAs associated with senescence driven by DNA damage and oxidative stress by analyzing changes in miRNA expression in *Ercc1*^−/−^ MEFs compared to the WT MEFs cultured to late passage in both high and low oxygen conditions. A subset of differentially expressed miRNAs (was found to be dysregulated in *Ercc1*^−/−^ MEFs driven to senescence compared to non-senescent *Ercc1*^−/−^ MEFs. Additionally, we demonstrate that several miRNAs differentially expressed in the *Ercc1*^−/−^ MEFs (miR-449a, miR-455*, miR-128, miR-497, miR-543, miR-450b-3p, miR-872 and miR-10b) were also dysregulated in liver tissues of both progeroid *Ercc1^−/Δ^* and old WT mice compared to young WT mice. We show that three of the above miRNAs (miR-449a, miR-455* and miR-128) were downregulated in kidney tissues from *Ercc1^−/Δ^*progeroid and WT old mice compared to the young mice. Finally, the regulator of miRNA biogenesis, Dicer, was significantly downregulated in late passage MEFs compared to early passage and in livers of old WT mice compared to young mice. The identified miRNAs in this study may play a critical role in staving off cellular senescence and aging.

## RESULTS

### Identification of senescence-associated miRNAs in *Ercc1*^−/−^ MEFs

We performed miRNA microarray analysis using RNA isolated from early and late passage WT and *Ercc1*^−/−^ MEFs to identify miRNAs that may contribute to cellular senescence. *Ercc1*^−/−^ MEFs grow slower than WT MEFs at 20% O_2_ and senesce prematurely by passage 7 (P7) [32, 44]. Growing MEFs at 3% O_2_ delays the onset of senescence due to decreased oxidative stress [41]. We compared the miRNA expression profiles of *Ercc1*^−/−^ and WT MEFs grown in low (3%) and high (20%) oxygen to passage 3 (early-passage, P3) and passage 7 (late-passage, P7) using mouse microRNA microarrays to discover miRNAs that correlate with senescence. We utilized Agilent mouse miRNA microarrays (V2), which probed for 627 total murine miRNAs. We focused on miRNAs that were significantly dysregulated, which was defined as ≥2-fold change in expression and *p* ≤.05. A comparison of the miRNA profiles of P3 (early passage) *Ercc1*^−/−^ and congenic WT MEFs grown along with further qRT-PCR validation showed minimal differences between the two samples regardless of oxygen tension (data not shown). This was not unexpected since the growth properties of *Ercc1*^−/−^ MEFs are not appreciably different from the WT MEFs at this early passage. We next compared the miRNA expression profiles of P7 *Ercc1*^−/−^ and WT MEFs of cells grown in either 20% O_2_ or 3% O_2_ to examine inherent changes in miRNA expression due to genotype. Comparison of P7 *Ercc1*^−/−^ and WT MEFs grown in 20% O_2_ identified one significantly upregulated miRNA, miR-467a, which was over-expressed 2.19 fold in P7 *Ercc1*^−/−^ MEFs compared to WT cells. MiR-467a may be upregulated as a result of a defect in DNA repair capacity. Additionally, we identified six downregulated miRNAs (miR-301a, miR-326, miR-455*, miR-497, miR-543 and miR-872) in late-passage P7 *Ercc1*^−/−^ MEFs compared to the WT MEFs grown in 3% O_2_ (Table 1). QRT-PCR analysis confirmed downregulation of these six miRNAs in *Ercc1*^−/−^ compared to WT MEFs, although there were differences in the fold-change downregulation based on the two approaches (Supplemental Figure 1).

**Table 1:** MiRNAs differentially expressed in *Ercc1*^−/−^ MEFs compared to WT MEFs grown at 3% O_2_

MicroRNA	Fold-change	*p*-value
mmu-miR-301a	−2.24	0.007
mmu-miR-543	−2.60	0.023
mmu-miR-326	−3.05	0.029
mmu-miR-455*	−3.23	0.020
mmu-miR-872	−6.72	0.044
mmu-miR-497	−6.87	0.002

DNA repair-deficient Ercc1^−/−^ primary MEFs were grown at 3% O_2_ to prevent cellular senescence. Congenic WT cells isolated from a littermate embryo were handled in parallel as a control. At passage 7, total RNA was isolated and miRNA expression measured by microarray. Significant changes were defined as ≥2-fold and p <.05 as determined by Welch's unpaired t test.

DNA repair-deficient Ercc1^−/−^ primary MEFs were grown at 20% O_2_ to induce cellular senescence. A parallel culture of the same cells was grown at 3% O_2_ to prevent sense-cence. At passage 7, total RNA was isolated and miRNA expression measured by hybridization-based microarray. Shown is the fold difference in expression for 20% versus 3% O_2_. Significant changes were defined as ≥2-fold and p <.05 as calculated via Welch's unpaired t test.

DNA repair-deficient Ercc1^−/−^ primary MEFs were grown at 3% O_2_. At passage 3 and 7, total RNA was isolated and miRNA expression measured by hybridization-based microarray. Shown is the fold difference in expression in P7 versus P3 cells. Significant changes were defined as ≥2-fold and p <.05 as determined by Welch's unpaired t test.

Three miRNAs (miR-450B-3p, miR-33 and miR-323-3p) were significantly upregulated in P7 *Ercc1*^−/−^ MEFs grown at 20% O_2_ compared to P7 *Ercc1*^−/−^ MEFs grown at 3% O_2_ (Table 2), which examined isogenic senescent vs. non-senescent cells. We next compared miRNA expression in P7 vs. P3 *Ercc1*^−/−^ MEFs grown in 3% (Table 3) or 20% O_2_ (Table 4), which identified miRNA dysregulated with sequential passaging of cells under different conditions of oxidative stress. Fourteen miRNAs were significantly upregulated and 22 downregulated in P7 *Ercc1*^−/−^ MEFs compared to P3 *Ercc1*^−/−^ MEFs grown at 3% O_2_ (Table 3). One miRNA, miR-24-2*, was upregulated and three miRNAs, miR-204, miR-218, miR-455* were downregulated in P7 *Ercc1*^−/−^ MEFs grown in 20% O_2_ compared to P3 *Ercc1*^−/−^ MEFs (Table 4). MiR-455* was downregulated in late passage cells compared to early passage in both analyses. Finally, we compared miRNA expression profiles of the most senescent cells in our analysis, P7 *Ercc1*^−/−^ MEFs grown at 20% O_2_, to the least senescent cells in our analysis, P3 WT MEFs grown at 3% O_2_. This revealed significant upregulation of one miRNA, miR-129-5p, which was increased 603-fold in *Ercc1*^−/−^ MEFs.

**Table 2:** MiRNAs differentially expressed in Ercc1^−/−^ MEFs grown at 20% vs. 3% O_2_

MicroRNA	Fold-change	*p-*value
mmu-miR-323-3p	2.14	0.003
mmu-miR-33	2.10	0.001
mmu-miR-450b-3p	2.06	0.015

**Table 3:** MiRNAs differentially expressed in late vs. early passage Ercc1^−/−^ MEFs

MicroRNA	Fold-change	*p*-value	MicroRNA	Fold-change	*p*-value
mmu-miR-671-5p	14.3	0.025	mmu-miR-29b*	−2.15	0.003
mmu-miR-1892	12.7	0.041	mmu-miR-449a	−2.24	0.043
mmu-miR-483	12.6	0.02	mmu-miR-455*	−2.77	0.004
mmu-miR-1894-3p	8.46	0.05	mmu-miR-340-3p	−2.96	0.036
mmu-miR-1895	7.28	0.039	mmu-miR-362-5p	−3.56	0.038
mmu-miR-680	6.54	0.023	mmu-miR-675-3p	−3.94	0.041
mmu-miR-721	6.43	0.029	mmu-miR-466a-3p	−3.95	0.037
mmu-miR-129-5p	5.06	0.046	mmu-miR-128	−4.41	0.047
mmu-miR-1906	3.82	0.006	mmu-miR-497	−5.16	0.048
mmu-miR-222	3.73	0.034	mmu-miR-362-3p	−5.23	0.012
mmu-miR-320	3.62	0.01	mmu-miR-192	−5.61	0.004
mmu-miR-290-5p	3.41	0.002	mmu-miR-496	−5.79	0.044
mmu-miR-22	3.27	0.023	mmu-miR-543	−6.81	0.023
mmu-miR-877	2.86	0.039	mmu-miR-30e*	−8.15	0.016
			mmu-miR-382*	−10.1	0.029
			mmu-miR-337-3p	−11.3	0.049
			mmu-miR-450b-3p	−12.4	0.014
			mmu-miR-872	−14.7	0.021
			mmu-miR-369-5p	−15.1	0.024
			mmu-miR-380-3p	−15.7	0.048
			mmu-miR-154*	−31.6	0.019
			mmu-miR-10b	−32.5	0.041

**Table 4:** MiRNAs differentially expressed in late vs. early passage Ercc1^−/−^ MEFs

MicroRNA	Fold-change	*p-*value
miR-24-2*	3.14	0.028
miR-455*	−3.98	0.036
miR-218	−10.3	0.032
miR-204	−13.1	0.022

DNA repair-deficient Ercc1^−/−^ primary MEFs were grown at 20% O_2_. At passage 3 and 7, total RNA was isolated and miRNA expression measured by hybridization-based microarray. Shown is the fold difference in expression in P7 versus P3 cells. Significant changes were defined as ≥2-fold and p <.05 as calculated via Welch's unpaired t test.

**Figure 1:**
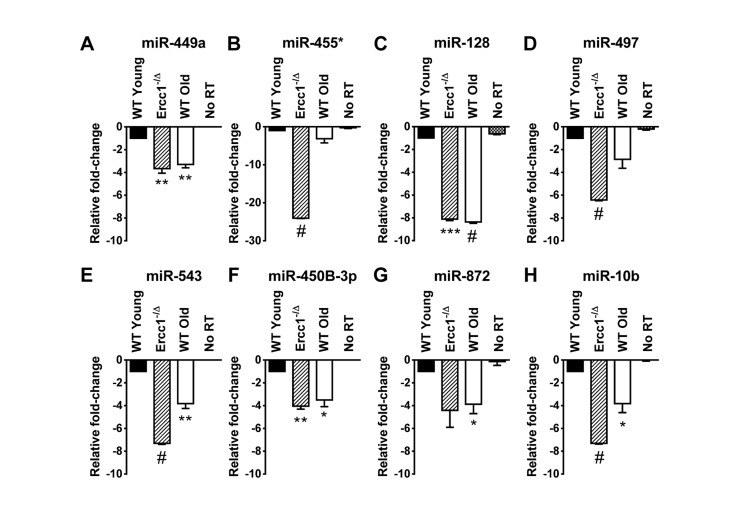
QRT-PCR quantification of miRNA identified as down-regulated in the liver of old WT mice and progeroid *Ercc1*^−/Δ^ mice compared to adult WT mice QRT-PCR analysis was performed on livers of WT young (20 weeks), *Ercc1*^−/Δ^ (20 weeks), and WT old mice (30 months). **(A)** miR-449a. **(B)** miR-455*. **(C)** miR-128. **(D)** miR-497. **(E)** miR-543. **(F)** miR-450b-3p. **(G)** miR-872. **(H)** miR-10b. All eight miRNAs were downregulated, most significantly, in *Ercc1*^−1Δ^ progeroid mice and WT old mice compared to WT young mice. No RT, no reverse transcriptase added. Three mouse livers are in each condition. The mean of three experimental replicates for each sample is graphed as relative to WT young samples, which were normalized to a value of -1. The standard deviation is plotted as error bars. *P*-values were calculated using Welch's t-tests and are indicated by * (p < .05), ** (p< .01), *** (p< .001) and # (p< .0001).

**Figure 2:**
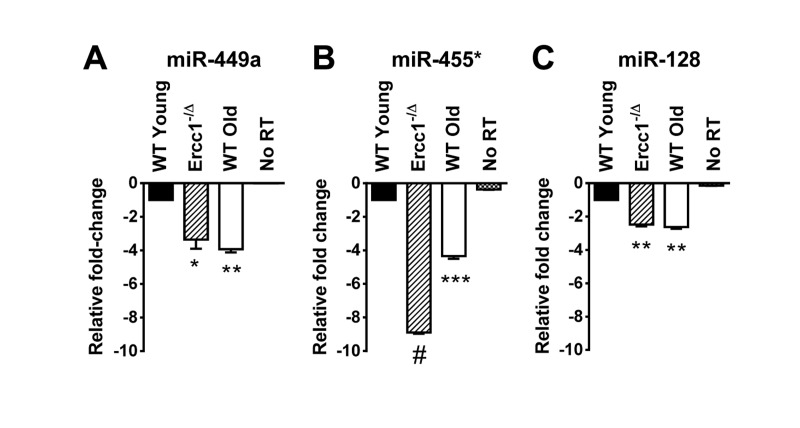
QRT-PCR quantification of miRNA identified as down-regulated in the kidney of old WT and progeroid *Ercc1*^−/Δ^ mice compared to adultWT kidney QRT-PCR analysis was performed on kidneys of WT young (20 weeks), *Ercc1*^−/Δ^ (20 weeks), and WT old mice (30 months). **(A)** miR-449a. **(B)** miR-455*. **(C)** miR-128. All three miRNAs identified in the microarray were significantly downregulated in kidney tissue of *Ercc1*^−/Δ^ progeroid mice and old mice compared to WT young mice. No RT, no reverse transcriptase added. Three mouse kidneys are in each condition. The mean of three experimental replicates for each sample is graphed as relative to WT young samples, which were normalized to a value of -1. The standard deviation is plotted as error bars. *P*-values were calculated comparing samples to WT Young using Welch's t-tests and are indicated by * (*p* < .05), ** (*p*< .01), *** (*p*< .001) and # (*p*<.0001).

For further studies, we selected sevenupregulated and ten downregulated miRNAs that were altered in P7 *Ercc1*^−/−^ MEFs compared to P3 MEFs grown at 3% O_2_ (Table 3) for validation by qRT-PCR analysis. This subset of 17 miRNAs was selected because they are known to have human homologs based on the miRBase database [45-48]. Of the seven upregulated miRNAs, three (miR-680, miR-320 and miR-22) were confirmed to be upregulated by qRT-PCR analysis (Supplemental Figure 2A). The other miRNAs showed either no significant change or were found to be downregulated as determined by qRT-PCR analysis. Ten down-regulated miRNAs were confirmed to be downregulated by qRT-PCR analysis (Supplemental Figure 2B). MiR-455*, miR-497 and miR-543 were significantly downregulated in P7 *Ercc1*^−/−^ MEFs compared to P7 WT MEFs (Table 1) and P7 versus P3 *Ercc1*^−/−^ MEFs (Table 3)grown at 3% O_2_, suggesting that these miRNAs may be dysregulated as a result of deficient DNA repair and/or sequential passaging.

We also confirmed that miR-467a was overexpressed in P7 *Ercc1*^−/−^ versus WT MEFs grown in 20% O_2_ usingqRT-PCR (Supplemental Figure 3A) and observed that it was also overexpressed in P7 versus P3 *Ercc1*^−/−^ MEFs grown in 20% O_2_, despite not appearing in our microarray data (Supplemental Figure 3B). Additionally, because miR-129-5p was the only miRNA dysregulated in the comparison of our most senescent to least senescent cells, we examined expression of this miRNA via qRT-PCR and confirmed that it is over-expressed in P7 *Ercc1*^−/−^ MEFs in 20% O_2_ compared to P3 WT MEFs in 3% O_2_ (Supplemental Figure 4).

Because the majority of miRNAs identified as dysregulated in ERCC1-depleted cells and tissue or old WT mice were downregulated rather than upregulated, we also measured expression of Dicer, which is required for miRNA biogenesis (Supplemental Figure 5A). Notably, levels of Dicer mRNA were significantly reduced in old WT mouse liver compared to tissue from young mice and in late passage MEFs compared to early passage (Supplemental Figure 5B). Therefore downregulation of numerous miRNAs with senescence and aging may arise as a consequence of reduced miRNA biosynthesis due to reduced Dicer expression.

### Senescence-associated miRNAs identified in *Ercc1*^−/−^ MEFs are downregulated in the livers of progeroid mice

The liver of 20 week-old progeroid *Ercc1*^−/Δ^ mice and 26 month-old WT mice show signs of profound cellular senescence [35]. This offers a unique opportunity to determine if the miRNA identified to correlate with senescence in vitro might play a role in senescence and aging in vivo. We analyzed the levels of 13 miRNAs confirmed to be dysregulated in P7 *Ercc1*^−/−^ MEFs compared to P3 *Ercc1*^−/−^ MEFs (miR-680, miR-320, miR-22, miR-449a, miR-455*, miR-675-3p, miR-128, miR-497, miR-543, miR-450b-3p, miR-872, miR-369-5p and miR-10b) in RNA samples prepared from the livers of WT young (20 weeks), the progeroid *Ercc1*^−/Δ^ mice, and WT old mice (30 months). Of the ten miRNAs downregulated in *Ercc1*^−/−^ MEFs, eight (miR-449a, miR-455*, miR-128, miR-497, miR-543, miR-450b-3p, miR-872 and miR-10b) were also down-regulated in both the progeroid and old WT mouse livers compared to the WT young (20 week) control mouse livers (Figure 1). The two remaining miRNAs downregulated in *Ercc1*^−/−^ MEFs, miR-369-5p and miR-675-3p, showed no expression changes in *Ercc1*^−/Δ^ mouse livers (data not shown). Three miRNAs (miR-680, miR-320, and miR-22) which were upregulated in P7 compared to P3 *Ercc1*^−/−^ MEFs (Table 4) as measured by microarray did not show upregulation in livers from progeroid and WT old mice compared to young WT controls as measured by qRT-PCR (data not shown).

### Three senescence-associated miRNAs identified in *Ercc1*^−/−^ MEFs are also downregulated in progeroid mice kidneys

In addition to severe liver abnormalities, ERCC1-deficient mice also develop significant renal dysfunction, as demonstrated by increased proteinuria and creatinine levels [32]. Renal histopathology is evident, including dilated renal tubules, nuclear abnormalities and fibrosis [33]. We examined RNA prepared from kidneys of young (20 weeks) *Ercc1*^−/Δ^ and WT mice, and old (30 months) WT mice to determine whether any of the aging-associated miRNAs we identified in this study were similarly dysregulated in the kidney tissue. Of the 8 downregulated miRNAs in *Ercc1*^−/Δ^ and WT old mouse liver compared to WT young mouse liver (Figure 1), three miRNAs (miR-449a, miR-455*, miR-128) were also downregulated in the kidneys of progeroid mice compared to WT young mice (Figure 2). Interestingly, these three miRNAs were also downregulated in the kidneys of old WT mice compared to the young WT mice (Figure 2), further strengthening the conclusion that these miRNAs may be aging-associated.

## DISCUSSION

This study is the first characterization of miRNA profiles in the mouse model of the XFE progeroid syndrome, enabling identification of miRNAs that are dysregulated as a consequence of cellular senescence and aging driven by endogenous DNA damage. We also compared the expression of differentially expressed miRNAs in liver and kidney tissues of *Ercc1*^−/Δ^ and WT old mice to those of young WT controls and found similarities between the progeroid and old mice. *Ercc1*^−/Δ^ mice have been established as a useful model for studying diseases of aging such as peripheral neuropathy, osteoporosis, intervertebral disk degeneration, and sarcopenia [49-51]. These studies showed that the rapid aging of *Ercc1*^−/Δ^ mice is very similar to that of normally aged mice. Herein we show that several downregulated miRNAs in the ERCC1-deficient mouse model of progeria are also down-regulated during normal murine aging. We focused on the liver and kidney since these organs are significantly affected in normal and accelerated aging and demonstrate accumulation of senescent cells [35]. Eight miRNAs (miR-449a, miR-455*, miR-128, miR-497, miR-543, miR-450b-3p, miR-872 and miR-10b) are significantly downregulated in the livers of progeroid *Ercc1*^−/Δ^ and naturally aged mice compared to young adult mice (Figure 1). The mice are genetically identical with the exception of the *Ercc1* mutation and in an f1 background (50:50 mix of C57Bl/6 and FVB). These data strongly support the conclusion that these miRNAs are dysregulated due to accelerated and natural aging. Three of these miRNAs (miR-128, miR-449a and miR-455*) were also downregulated in the kidneys of progeroid and WT old mouse compared to the young WT mouse kidneys (Figure 2). Both the liver and kidney of progeroid ERCC1-deficient mice and old WT mice show aging-related functional and degenerative changes as well as profound cellular senescence [35, 52]. Consistently, the same miRNAs were detected as downregulated in late passage *Ercc1*^−/−^ MEFs (Table 3). The combination of in vitro and in vivo data strongly point to the conclusion that these miRNAs play a role in driving cellular senescence and aging, or are powerful biomarkers of these physiological changes.

Previously confirmed gene targets of the miRNAs identified in this study that are linked to cellular senescence and aging (miR-449a, miR-455*, miR-128, miR-497, miR-543, miR-450b-3p, miR-872 and miR-10b) are listed in Supplemental Table S1. Several of the 43 gene targets such as Sirt1, Bcl2, Sod1, and Myc are involved in cell cycle control and cellular stress responses. Genes associated with cellular senescence as well as p53 downstream targets (Ccnd1, Cdk6) are also target genes. This list of potential miRNA targets is consistent with a possible role of these miRNAs in cellular senescence and aging.

Three miRNAs (miR-128, miR-449a and miR-455*) are downregulated in late passage MEFs as well as liver and kidney tissues of both progeroid *Ercc1*^−/Δ^ and WT old mice. MiR-128 is known to promote cell survival [53]. MiR-449a targets critical cell cycle regulatory proteins such as Cyclin D1 [54, 55]. Interestingly, it was shown that high Cyclin D1 expression is observed in senescent cells exhibiting a “pseudo-DNA damage response” and that Cyclin D1 is overexpressed in fibroblasts undergoing replicative senescence [56, 57]. These observations are in line with our results that miR-449a is downregulated in senescent cells. The transcription factor, Hnf4a, is a target of miR-449a in liver cells [58]. Hnf4a is essential to liver development and maintenance, and when suppressed, it can cause epigenetic changes that lead to increased incidence of hepatocellular cancer [59 Reduced expression of miR-449 in aging liver would increase Hnf4a expression, possibly preventing hepatocyte transformation.

MiR-10b, which we detected as significantly downregulated in senescent MEFs and aged liver, is upregulated in breast cancer and gliomas and its expression closely correlates with tumor cell metastatic potential [60]. It is possible that dysregulation of one or more of the above miRNAs may result in reduced cell growth and increased cellular senescence through their regulation of target genes. Less is known about the cellular targets and function of miR-450b-3p, miR-455*, miR-543 and miR-872 (all downregulated in our studies).

Since the majority of differentially expressed miRNA identified in this study were downregulated in senescence and aging, we examined Dicer expression and found it to be downregulated in senescent MEFs (Supplemental Figure 5A). Dicer protein and mRNA transcript were significantly downregulated in normally aged mice white adipose tissues and elderly human preadipocytes [61]. A recent study showed an approximately two-fold downregulation of Dicer expression in senescent human umbilical vein endothelial cells compared to early passage cells, further implicating a role for Dicer downregulation in senescence and aging [62].

The *Zmpste24*^−/−^ mouse model of Hutchinson-Gilford progeria demonstrates miR-29b is upregulated in liver and muscle of both progeroid and normally aged mice [43] MiR-29b is a positive regulator of the p53-mediated DNA damage response. The paper by Ugalde et al. was the first to use a progeroid mouse models in identifying miRNAs that regulate DNA repair processes and senescence. Another study using murine and *Caenorhabditis elegans* models of the progeroid disease Werner Syndrome identified miR-124 as modulator of reactive oxygen species and ATP production [63]. Our study further underscores the utility of rapid aging mouse models to study miRNA dysregulation in aging and cellular senescence. We have used a progeroid model of endogenous DNA damage accumulation to identify miRNA dysregulation common to both the Ercc1-deficient mouse model of progeria and normal mouse aging in liver and kidney tissues.

In summary, we identified several miRNAs that are similarly dysregulated in senescent primary MEFs and senescent tissues of progeroid and naturally aged mice (miR-449a, miR-455*, miR-128, miR-497, miR-543, miR-450b-3p, miR-872 and miR-10b). We have shown that Dicer expression is downregulated in senescence induced by genotoxic stress, and that the miRNA downregulation that we observe in this study could be a consequence of global miRNA downregulation. These miRNA are promising as biomarkers of aging and factors that may be critical for preventing cell senescence and aging-related degenerative changes in response to genotoxic stress.

## EXPERIMENTAL PROCEDURES

### Animal Care and Experimentation

All experiments involving mouse tissues and cells were approved by the University of Pittsburgh Institutional Animal Care and Use Committee and were in accordance with NIH guidelines for humane care of animals. *Ercc1*^−/−^ and *Ercc1*^−/Δ^ mice were bred and genotyped as previously described [39].ll mice used for tissue miRNA qRT-PCR analysis were in a f1 mixed genetic background (FVB/n:C57Bl/6). Twenty-week old progeroid *Ercc1*^−/Δ^ mice along with age-matched WT littermates with aged (30 month) WT mice were euthanized by CO_2_ inhalation and tissues were excised and flash-frozen in liquid nitrogen. Three mice of each genotype were used for subsequent qRT-PCR analysis. The livers and kidneys were homogenized with a hand-held homogenizer (Omni International, Kennesaw, GA, USA) and RNA was extracted using the Ultraspec RNA Isolation System (Biotecx, Houston, TX, USA). Following isolation, RNA quantity was measured using a Nanodrop (Thermo Fisher Scientific Inc., Waltham, MA, USA). RNA quality was assessed by formaldehyde-agarose gel electrophoresis.

### Primary Mouse Embryonic Fibroblasts

The following primary cells were used: wild-type (WT) isogenic mouse embryonic fibroblasts (MEFs) and *Ercc1*^−/−^ (KO) MEFs derived from 13.5-day embryos with a 50:50 C57Bl/6:FVB/n background. Cells were serially passaged at either 3% or 20% O_2_ to passage 3 or passage 7, pelleted and total RNA extracted using the Ultraspec RNA Isolation System. Two independent cell lines were used for microarray and qRT-PCR analysis.

### MicroRNA Microarray

MicroRNA microarray studies were performed using 100 nanograms of total RNA obtained from WT and *Ercc1*^−/−^ MEFs grown to P3 and P7 in 3% or 20% O_2_. Two independent cell lines were used for each sample and both cell lines were analyzed via microarray. We used the Agilent mouse miRNA microarrays (V2) (Agilent Technologies, Santa Clara, CA, USA) according to the manufacturer's instructions. Each microarray contains sixteen to twenty oligonucleotide probes for each of the 627 mouse miRNAs and 39 mouse viral miRNAs based on the Sanger database version 12. The array contains 281 positive controls consisting of high-signal endogenous mouse-specific probes, and 434 negative controls consisting of random sequences that have low signal and poor hybridization to mouse RNA. RNA was isolated from MEFs using the Ultraspec RNA Isolation System, dephosphorylated with calf intestinal alkaline phosphatase, and then denatured with dimethyl sulfoxide. The 3' ends were then ligated to a Cyanine3-pCp molecule using T4 RNA ligase. Labeled RNA was purified using a MicroBio-spin 6 column containing Bio-Gel P-6 in Tris buffer (Bio-Rad Laboratories, Inc. Hercules, CA, USA). The labeled RNA samples were hybridized to Agilent microarray slides at 55°C for 20 hours. Following hybridization, the slides were washed with Agilent-supplied Gene Expression Wash Buffers 1 and 2. Slides were immediately scanned with an Agilent Microarray Scanner.

### Microarray Statistical Analysis

After scanning, images were processed using Agilent's Feature Extraction Software, version 9.5.3. Extracted data were exported into Agilent's GeneSpring GX version 10 software and microarray data was log_2_ transformed and normalized to the mean of each array. An unpaired t-test with unequal variance was used to identify differentially expressed miRNAs (P< 0.05). We selected microRNAs with 2-fold or greater changes for further study. Microarray experiments conform to Minimum Information About a Microarray Experiment (MAIME) guidelines and a full data set has been submitted to the National Center for Biotechnology Information (NCBI) Gene Expression Omnibus database (GEO).

### Real-Time Quantitative Reverse-Transcriptase Poly-merase Chain Reaction (qRT-PCR) analysis

QRT-PCR analysis was performed on total RNA prepared by Ultraspec RNA isolation by using a two-step individual Mature TaqmanŴ MicroRNA Assays kit (Applied Biosystems, Foster City, CA, USA) and the Real-Time Thermocycler iQ5 (Bio-Rad, Hercules, CA, USA). All qRT-PCR assays were performed according to manufacturer's instructions and miRNA expression levels were normalized to snoRNA135. For all experiments, two independent cell lines were used and all assays were performed in triplicate. Relative expression was calculated using the 2^−ΔΔCT^ method [64]. Welch's unpaired *t* test with 95% confidence intervals was performed for statistical analysis of all qRT-PCR experiments using Prism software (GraphPad Software, Inc., La Jolla, CA, USA).

Dicer expression in primary MEFs and mouse livers was quantified via qRT-PCR using the iScript One-Step RT-PCR Kit with SYBR Green (BioRad) in accordance with the manufacturer's instructions. Dicer mRNA was amplified using the forward primer sequence 5'-GGAA GCAGCCAACAAAAGAG- 3' and the reverse primer 5'-TGAGGGTTTTCTCTGCGTCT-3', amplifying a 145-bp region. Dicer mRNA levels were normalized to the glyceraldehyde-3-phosphate dehydrogenase (GAPDH) gene, using the forward primer 5'-AACTTTGGCATT GTGGAAGG-3' and the reverse primer5'-GGATGCAGGGATGATGTTCT-3', amplify-ing a 132-bp region. DNase I-treated total RNA (1 Ɓg) was used for each reaction, and all the reactions were performed in triplicate. Relative Dicer mRNA expression was calculated using 2^−ΔΔCT^ values [64]. Welch's unpaired *t* test with 95% confidence intervals was performed for statistical analysis of all qRT-PCR experiments using Prism software (GraphPad Software, Inc., La Jolla, CA, USA).

## SUPPLEMENTAL DATA



## References

[R1] Wu L, Belasco JG (2008). Let me count the ways: mechanisms of gene regulation by miRNAs and siRNAs. Molecular cell.

[R2] Rivas FV, Tolia NH, Song JJ, Aragon JP, Liu J, Hannon GJ, Joshua-Tor L (2005). Purified Argonaute2 and an siRNA form recombinant human RISC. Nat Struct Mol Biol.

[R3] Kim VN, Han J, Siomi MC (2009). Biogenesis of small RNAs in animals. Nature reviews Molecular cell biology.

[R4] Iorio MV, Croce CM (2012). Causes and consequences of microRNA dysregulation. Cancer journal (Sudbury, Mass).

[R5] Rodier F, Campisi J (2011). Four faces of cellular senescence. J Cell Biol.

[R6] Martens UM, Chavez EA, Poon SS, Schmoor C, Lansdorp PM (2000). Accumulation of short telomeres in human fibroblasts prior to replicative senescence. Experimental cell research.

[R7] Serrano M, Lin AW, McCurrach ME, Beach D, Lowe SW (1997). Oncogenic ras provokes premature cell senescence associated with accumulation of p53 and p16INK4a. Cell.

[R8] Di Leonardo A, Linke SP, Clarkin K, Wahl GM (1994). DNA damage triggers a prolonged p53-dependent G1 arrest and long-term induction of Cip1 in normal human fibroblasts. Genes … development.

[R9] Chang BD, Broude EV, Fang J, Kalinichenko TV, Abdryashitov R, Poole JC, Roninson IB (2000). p21Waf1/Cip1/Sdi1-induced growth arrest is associated with depletion of mitosis-control proteins and leads to abnormal mitosis and endoreduplication in recovering cells. Oncogene.

[R10] Pospelova TV, Demidenko ZN, Bukreeva EI, Pospelov VA, Gudkov AV, Blagosklonny MV (2009). Pseudo-DNA damage response in senescent cells. Cell cycle.

[R11] Demidenko ZN, Blagosklonny MV (2008). Growth stimulation leads to cellular senescence when the cell cycle is blocked. Cell cycle.

[R12] Rodier F, Coppe JP, Patil CK, Hoeijmakers WA, Munoz DP, Raza SR, Freund A, Campeau E, Davalos AR, Campisi J (2009). Persistent DNA damage signalling triggers senescence-associated inflammatory cytokine secretion. Nat Cell Biol.

[R13] Sharpless NE (2004). Ink4a/Arf links senescence and aging. Experimental gerontology.

[R14] Baker DJ, Wijshake T, Tchkonia T, LeBrasseur NK, Childs BG, van de Sluis B, Kirkland JL, van Deursen JM (2011). Clearance of p16Ink4a-positive senescent cells delays ageing-associated disorders. Nature.

[R15] Bhaumik D, Scott GK, Schokrpur S, Patil CK, Orjalo AV, Rodier F, Lithgow GJ, Campisi J (2009). MicroRNAs miR-146a/b negatively modulate the senescence-associated inflammatory mediators IL-6 and IL-8. Aging.

[R16] Xu D, Takeshita F, Hino Y, Fukunaga S, Kudo Y, Tamaki A, Matsunaga J, Takahashi RU, Takata T, Shimamoto A, Ochiya T, Tahara H (2011). miR-22 represses cancer progression by inducing cellular senescence. J Cell Biol.

[R17] Abdelmohsen K, Srikantan S, Tominaga K, Kang MJ, Yaniv Y, Martindale JL, Yang X, Park SS, Becker KG, Subramanian M, Maudsley S, Lal A, Gorospe M (2012). Growth inhibition by miR-519 via multiple p21-inducing pathways. Mol Cell Biol.

[R18] Mancini M, Saintigny G, Mahe C, Annicchiarico-Petruzzelli M, Melino G, Candi E (2012). MicroRNA-152 and -181a participate in human dermal fibroblasts senescence acting on cell adhesion and remodeling of the extra-cellular matrix. Aging.

[R19] Mudhasani R, Zhu Z, Hutvagner G, Eischen CM, Lyle S, Hall LL, Lawrence JB, Imbalzano AN, Jones SN (2008). Loss of miRNA biogenesis induces p19Arf-p53 signaling and senescence in primary cells. J Cell Biol.

[R20] Rizzo M, Evangelista M, Simili M, Mariani L, Pitto L, Rainaldi G (2011). Immortalization of MEF is characterized by the deregulation of specific miRNAs with potential tumor suppressor activity. Aging.

[R21] Brown-Borg HM, Borg KE, Meliska CJ, Bartke A (1996). Dwarf mice and the ageing process. Nature.

[R22] Kimura KD, Tissenbaum HA, Liu Y, Ruvkun G (1997). daf-2, an insulin receptor-like gene that regulates longevity and diapause in Caenorhabditis elegans. Science.

[R23] Flurkey K, Papaconstantinou J, Harrison DE (2002). The Snell dwarf mutation Pit1(dw) can increase life span in mice. Mech Ageing Dev.

[R24] Maes OC, An J, Sarojini H, Wang E (2008). Murine microRNAs implicated in liver functions and aging process. Mech Ageing Dev.

[R25] Inukai S, de Lencastre A, Turner M, Slack F (2012). Novel microRNAs differentially expressed during aging in the mouse brain. PLoS One.

[R26] Bates DJ, Li N, Liang R, Sarojini H, An J, Masternak MM, Bartke A, Wang E (2010). MicroRNA regulation in Ames dwarf mouse liver may contribute to delayed aging. Aging Cell.

[R27] Li X, Khanna A, Li N, Wang E (2011). Circulatory miR34a as an RNAbased, noninvasive biomarker for brain aging. Aging.

[R28] Khanna A, Muthusamy S, Liang R, Sarojini H, Wang E (2011). Gain of survival signaling by down-regulation of three key miRNAs in brain of calorie-restricted mice. Aging.

[R29] ElSharawy A, Keller A, Flachsbart F, Wendschlag A, Jacobs G, Kefer N, Brefort T, Leidinger P, Backes C, Meese E, Schreiber S, Rosenstiel P, Franke A (2012). Genome-wide miRNA signatures of human longevity. Aging Cell.

[R30] Gombar S, Jung HJ, Dong F, Calder B, Atzmon G, Barzilai N, Tian XL, Pothof J, Hoeijmakers JH, Campisi J, Vijg J, Suh Y (2012). Comprehensive microRNA profiling in B-cells of human centenarians by massively parallel sequencing. BMC genomics.

[R31] Sheinerman KS, Tsivinsky VG, Crawford F, Mullan MJ, Abdullah L, Umansky SR (2012). Plasma microRNA biomarkers for detection of mild cognitive impairment. Aging.

[R32] Niedernhofer LJ, Garinis GA, Raams A, Lalai AS, Robinson AR, Appeldoorn E, Odijk H, Oostendorp R, Ahmad A, van Leeuwen W, Theil AF, Vermeulen W, van der Horst GT (2006). A new progeroid syndrome reveals that genotoxic stress suppresses the somatotroph axis. Nature.

[R33] Lawrence NJ, Sacco JJ, Brownstein DG, Gillingwater TH, Melton DW (2008). A neurological phenotype in mice with DNA repair gene Ercc1 deficiency. DNA repair.

[R34] Dollé MET, Kuiper RV, Roodbergen M, Robinson J, Vlugt Sd, Wijnhoven SWP, Beems RB, Fonteyne Ldl, With Pd, Pluijm Ivd, Niedernhofer LJ, Hasty P, Vijg J (2011). Broad segmental progeroid changes in short-lived Ercc1 −/ … Delta;7 mice.

[R35] Gregg SQ, Gutierrez V, Rasile Robinson A, Woodell T, Nakao A, Ross MA, Michalopoulos GK, Rigatti L, Rothermel CE, Kamileri I, Garinis GA, Beer Stolz D, Niedernhofer LJ (2012). A mouse model of accelerated liver aging caused by a defect in DNA repair. Hepatology.

[R36] Borgesius NZ, de Waard MC, van der Pluijm I, Omrani A, Zondag GC, van der Horst GT, Melton DW, Hoeijmakers JH, Jaarsma D, Elgersma Y (2011). Accelerated age-related cognitive decline and neurodegeneration, caused by deficient DNA repair. The Journal of neuroscience : the official journal of the Society for Neuroscience.

[R37] Moggs JG, Yarema KJ, Essigmann JM, Wood RD (1996). Analysis of incision sites produced by human cell extracts and purified proteins during nucleotide excision repair of a 1,3-intrastrand d(GpTpG)-cisplatin adduct. J Biol Chem.

[R38] Bhagwat N, Olsen AL, Wang AT, Hanada K, Stuckert P, Kanaar R, D'Andrea A, Niedernhofer LJ, McHugh PJ (2009). XPF-ERCC1 participates in the Fanconi anemia pathway of cross-link repair. Mol Cell Biol.

[R39] Ahmad A, Robinson AR, Duensing A, van Drunen E, Beverloo HB, Weisberg DB, Hasty P, Hoeijmakers JH, Niedernhofer LJ (2008). ERCC1-XPF endonuclease facilitates DNA double-strand break repair. Mol Cell Biol.

[R40] Wang J, Clauson CL, Robbins PD, Niedernhofer LJ, Wang Y (2012). The oxidative DNA lesions 8,5'-cyclopurines accumulate with aging in a tissue-specific manner. Aging Cell.

[R41] Parrinello S, Samper E, Krtolica A, Goldstein J, Melov S, Campisi J (2003). Oxygen sensitivity severely limits the replicative lifespan of murine fibroblasts. Nat Cell Biol.

[R42] Weeda G, Donker I, de Wit J, Morreau H, Janssens R, Vissers CJ, Nigg A, van Steeg H, Bootsma D, Hoeijmakers JH (1997). Disruption of mouse ERCC1 results in a novel repair syndrome with growth failure, nuclear abnormalities and senescence. Curr Biol.

[R43] Ugalde AP, Ramsay AJ, de la Rosa J, Varela I, Marino G, Cadinanos J, Lu J, Freije JM, Lopez-Otin C (2011). Aging and chronic DNA damage response activate a regulatory pathway involving miR-29 and p53. EMBO J.

[R44] Tilstra JS, Robinson AR, Wang J, Gregg SQ, Clauson CL, Reay DP, Nasto LA, St Croix CM, Usas A, Vo N, Huard J, Clemens PR, Stolz DB (2012). NF-kappaB inhibition delays DNA damage-induced senescence and aging in mice. J Clin Invest.

[R45] Griffiths-Jones S (2004). The microRNA Registry. Nucleic Acids Research.

[R46] Griffiths-Jones S, Grocock RJ, van Dongen S, Bateman A, Enright AJ (2006). miRBase: microRNA sequences, targets and gene nomenclature. Nucleic Acids Research.

[R47] Griffiths-Jones S, Saini HK, van Dongen S, Enright AJ (2008). miRBase: tools for microRNA genomics. Nucleic Acids Research.

[R48] Kozomara A, Griffiths-Jones S (2011). miRBase: integrating microRNA annotation and deep-sequencing data. Nucleic Acids Research.

[R49] Goss JR, Stolz DB, Robinson AR, Zhang M, Arbujas N, Robbins PD, Glorioso JC, Niedernhofer LJ (2011). Premature aging-related peripheral neuropathy in a mouse model of progeria. Mech Ageing Dev.

[R50] Vo N, Seo HY, Robinson A, Sowa G, Bentley D, Taylor L, Studer R, Usas A, Huard J, Alber S, Watkins SC, Lee J, Coehlo P (2010). Accelerated aging of intervertebral discs in a mouse model of progeria. Journal of orthopaedic research : official publication of the Orthopaedic Research Society.

[R51] Lavasani M, Robinson AR, Lu A, Song M, Feduska JM, Ahani B, Tilstra JS, Feldman CH, Robbins PD, Niedernhofer LJ, Huard J (2012). Muscle-derived stem/progenitor cell dysfunction limits healthspan and lifespan in a murine progeria model. Nature communications.

[R52] Selfridge J, Hsia KT, Redhead NJ, Melton DW (2001). Correction of liver dysfunction in DNA repair-deficient mice with an ERCC1 transgene. Nucleic Acids Res.

[R53] Papagiannakopoulos T, Friedmann-Morvinski D, Neveu P, Dugas JC, Gill RM, Huillard E, Liu C, Zong H, Rowitch DH, Barres BA, Verma IM, Kosik KS (2012). Pro-neural miR-128 is a glioma tumor suppressor that targets mitogenic kinases. Oncogene.

[R54] Noonan EJ, Place RF, Basak S, Pookot D, Li LC (2010). miR-449a causes Rb-dependent cell cycle arrest and senescence in prostate cancer cells. Oncotarget.

[R55] Bou Kheir T, Futoma-Kazmierczak E, Jacobsen A, Krogh A, Bardram L, Hother C, Gronbaek K, Federspiel B, Lund AH, Friis-Hansen L (2011). miR-449 inhibits cell proliferation and is down-regulated in gastric cancer. Molecular cancer.

[R56] Leontieva OV, Lenzo F, Demidenko ZN, Blagosklonny MV (2012). Hyper-mitogenic drive coexists with mitotic incompetence in senescent cells. Cell cycle.

[R57] Lucibello FC, Sewing A, Brusselbach S, Burger C, Muller R (1993). Deregulation of cyclins D1 and E and suppression of cdk2 and cdk4 in senescent human fibroblasts. Journal of cell science.

[R58] Ramamoorthy A, Li L, Gaedigk A, Bradford LD, Benson EA, Flockhart DA, Skaar TC (2012). In silico and in vitro identification of microRNAs that regulate hepatic nuclear factor 4alpha expression. Drug metabolism and disposition: the biological fate of chemicals.

[R59] Hatziapostolou M, Polytarchou C, Aggelidou E, Drakaki A, Poultsides GA, Jaeger SA, Ogata H, Karin M, Struhl K, Hadzopoulou-Cladaras M, Iliopoulos D (2011). An HNF4alpha-miRNA inflammatory feedback circuit regulates hepatocellular oncogenesis. Cell.

[R60] Gabriely G, Yi M, Narayan RS, Niers JM, Wurdinger T, Imitola J, Ligon KL, Kesari S, Esau C, Stephens RM, Tannous BA, Krichevsky AM (2011). Human glioma growth is controlled by microRNA-10b. Cancer Res.

[R61] Mori MA, Raghavan P, Thomou T, Boucher J, Robida-Stubbs S, Macotela Y, Russell SJ, Kirkland JL, Blackwell TK, Kahn CR (2012). Role of microRNA processing in adipose tissue in stress defense and longevity. Cell metabolism.

[R62] Dellago H, Preschitz-Kammerhofer B, Terlecki-Zaniewicz L, Schreiner C, Fortschegger K, Chang MW, Hackl M, Monteforte R, Kuhnel H, Schosserer M, Gruber F, Tschachler E, Scheideler M (2013). High levels of oncomiR-21 contribute to the senescence-induced growth arrest in normal human cells and its knock-down increases the replicative lifespan. Aging Cell.

[R63] Dallaire A, Garand C, Paquel ER, Mitchell SJ, de Cabo R, Simard MJ, Lebel M (2012). Down regulation of miR-124 in both Werner syndrome DNA helicase mutant mice and mutant Caenorhabditis elegans wrn-1 reveals the importance of this microRNA in accelerated aging. Aging.

[R64] Livak KJ, Schmittgen TD (2001). Analysis of relative gene expression data using real-time quantitative PCR and the 2(-Delta Delta C(T)) Method. Methods.

[R65] Zhu Y, Yu F, Jiao Y, Feng J, Tang W, Yao H, Gong C, Chen J, Su F, Zhang Y, Song E (2011). Reduced miR-128 in breast tumor-initiating cells induces chemotherapeutic resistance via Bmi-1 and ABCC5. Clinical cancer research : an official journal of the American Association for Cancer Research.

[R66] Adlakha YK, Saini N (2011). MicroRNA-128 downregulates Bax and induces apoptosis in human embryonic kidney cells. Cellular and molecular life sciences : CMLS.

[R67] Donzelli S, Fontemaggi G, Fazi F, Di Agostino S, Padula F, Biagioni F, Muti P, Strano S, Blandino G (2012). MicroRNA-128-2 targets the transcriptional repressor E2F5 enhancing mutant p53 gain of function. Cell Death Differ.

[R68] Guidi M, Muinos-Gimeno M, Kagerbauer B, Marti E, Estivill X, Espinosa-Parrilla Y (2010). Overexpression of miR-128 specifically inhibits the truncated isoform of NTRK3 and upregulates BCL2 in SH-SY5Y neuroblastoma cells. BMC molecular biology.

[R69] Shi ZM, Wang J, Yan Z, You YP, Li CY, Qian X, Yin Y, Zhao P, Wang YY, Wang XF, Li MN, Liu LZ, Liu N (2012). MiR-128 inhibits tumor growth and angiogenesis by targeting p70S6K1. PLoS One.

[R70] Lize M, Klimke A, Dobbelstein M (2011). MicroRNA-449 in cell fate determination. Cell cycle.

[R71] Yadav S, Pandey A, Shukla A, Talwelkar SS, Kumar A, Pant AB, Parmar D (2011). miR-497 and miR-302b regulate ethanol-induced neuronal cell death through BCL2 protein and cyclin D2. J Biol Chem.

[R72] Guo ST, Jiang CC, Wang GP, Li YP, Wang CY, Guo XY, Yang RH, Feng Y, Wang FH, Tseng HY, Thorne RF, Jin L, Zhang XD (2013). MicroRNA-497 targets insulin-like growth factor 1 receptor and has a tumour suppressive role in human colorectal cancer. Oncogene.

[R73] Zheng D, Radziszewska A, Woo P (2012). MicroRNA 497 modulates interleukin 1 signalling via the MAPK/ERK pathway. FEBS letters.

[R74] Haga CL, Phinney DG (2012). MicroRNAs in the imprinted DLK1-DIO3 region repress the epithelial-to-mesenchymal transition by targeting the TWIST1 protein signaling network. J Biol Chem.

[R75] Papaioannou MD, Lagarrigue M, Vejnar CE, Rolland AD, Kuhne F, Aubry F, Schaad O, Fort A, Descombes P, Neerman-Arbez M, Guillou F, Zdobnov EM, Pineau C (2011). Loss of Dicer in Sertoli cells has a major impact on the testicular proteome of mice. Molecular … cellular proteomics : MCP.

[R76] Friedman RC, Farh KK, Burge CB, Bartel DP (2009). Most mammalian mRNAs are conserved targets of microRNAs. Genome research.

