# Supplementary material for: Identification of microRNAs dysregulated in cellular senescence driven by endogenous genotoxic stress
Source: Aging (Albany NY). 2013 Jun 28;5(6):460–73. doi: 10.18632/aging.100571 (PMC3824412; doi:10.18632/aging.100571)
Supplement: Supplementary file 1 [file aging-05-460-s001.pdf]

SUPPLEMENTAL DATA

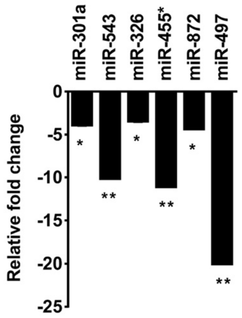

Supplemental Figure 1. QRT-PCR confirmation of downregulation of six miRNAs identified from the microarray experiments in *Ercc1*$^{-/-}$ MEFs, compared to WT MEFs. MiRNA microarray data (Table 1) was validated by qRT-PCR analysis of *Ercc1-/-* MEFs at P7 compared to WT MEFs at P7, grown to 3% O_2. WT MEFs at P7 were assigned a value of -1. Six miRNAs identified in the microarray were confirmed to be downregulated by qRT-PCR. MiR-455* refers to the less abundant miRNA product derived from the miR-455 stem-loop precursor. *P*-values were calculated using Welch's t-tests and are indicated by * (*p*< .001) and ** (*p*< .0001).

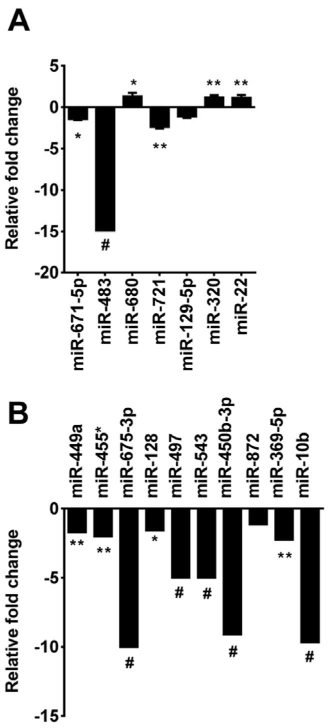

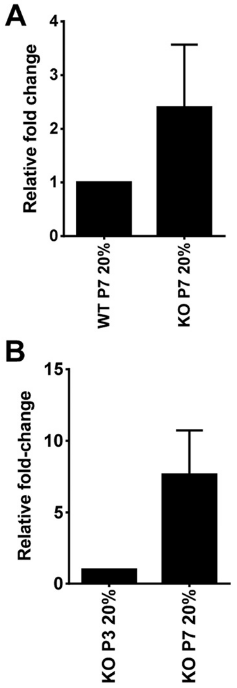

Supplemental Figure 2. QRT-PCR validation of miRNAs identified from the microarray analysis of late passage *Ercc1*$^{-/-}$ MEFs normalized to early passage *Ercc1*$^{-/-}$ MEFs. (A) Three miRNAs identified identified as being upregulated in late *Ercc1*$^{-/-}$ MEFs compared to early passage via microarray (Table 3) were confirmed to be upregulated by qRT-PCR. QRT-PCR expression values are relative to *Ercc1*$^{-/-}$ passage 3 samples, which were normalized to a value of either 1 or -1. **(B)** Ten downregulated miRNAs in P7 *Ercc1*$^{-/-}$ MEFs from the microarray analysis (Table 3) were confirmed to be downregulated by qRT-PCR. MiR-455* refers to the less abundant miRNA product derived from the miR-455 stem-loop precursor. Expression values are relative to *Ercc1*$^{-/-}$ passage 3 samples, which were normalized to a value of -1. *P*-values for qRT-PCR data were calculated using Welch's t-tests and are indicated by * (*p* < .05), ** (*p*< .01) and # (*p*< .0001).

Supplemental Figure 3. QRT-PCR expression of miR-467a in Passage 3 *Ercc1*$^{-/-}$ MEFs, Passage 7 WT MEFs and Passage 7 *Ercc1*$^{-/-}$ MEFs grown in 20% O2. (A) MiR-467a is upregulated in *Ercc1*$^{-/-}$ MEFs (KO) grown to Passage 7 (P7) in 20% O2 compared to WT P7 MEFs grown to 20% O_2. QRT-PCR expression values are relative to WT P7 samples, which were normalized to a value of 1. **(B)** MiR-467a is upregulated in *Ercc1*$^{-/-}$ MEFs (KO) grown to Passage 7 (P7) in 20% O2 compared to *Ercc1*$^{-/-}$ MEFs (KO) grown to Passage 3 in 20% O2. Expression values are relative to *Ercc1*$^{-/-}$ passage 3 samples, which were normalized to a value of 1. All experiments were performed in triplicate and the standard deviation is plotted as error bars.

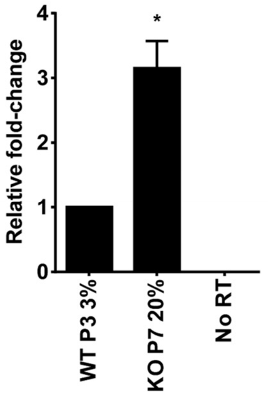

Supplemental Figure 4. QRT-PCR expression of miR-129-5p in Passage 3 WT MEFs grown in 3% O_2 and Passage 7 *Ercc1*$^{-/-}$ MEFs grown in 20% O_2. (A) MiR-129-5p is upregulated in late passage senescent *Ercc1*$^{-/-}$ MEFs (KO P7) grown in 20% O_2 compared to WT Passage 3 (P3) MEFs grown in 3% O2,which are the least senescent cells that we examined in our microarray experiments. QRT-PCR expression values are relative to WT passage 3 MEFs, which were normalized to a value of 1. All experiments were performed in triplicate and the standard deviation is plotted as error bars. *P*-values for qRT-PCR data were calculated using Welch's t-tests and are indicated by * (*p* < .05).

Supplemental Table S1. Experimentally validated target genes for miRNAs identified in this study. 3'-UTR homology between human and mouse gene orthologs was etermined using Targetscan 6.2 [76].

miRNA	Target gene(s)	References
miR-10b	Bcl2l11, Hoxd10, Tfap2c	[60]
miR-128	Abcc5, Bax, Bmi1, E2F3a, E2f5, Hoxa10, Ntrk3, Reln, Ret, Rps6kb1	[65-69]
miR-449a	Bcl2, Ccnd1, Ccne2, Cdc25a, Cdk6, Dll1, E2f2, E2f3, E2f5, Gmnn, Hdac1, Hnf4a, Lef1, Met, Myc, Mycn, Notch1, Sirt1	[70]
miR-450b-3p	No confirmed mRNA targets	
miR-455*	No confirmed mRNA targets	
miR-497	Bcl2, Bcl2l2, Ccnd1, Ccnd2, Igf1r, Map2k1, Raf1	[71-73]
miR-543	Twist1	[74]
miR-872	Sod1	[75]

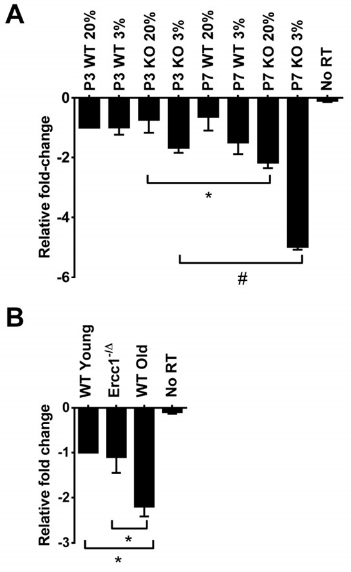

Supplemental Figure 5. QRT-PCR of Dicer mRNA in primary MEFs and in liver tissues shows reduced expression in senescence and aging. (A) Dicer mRNA is downregulated in Passage 7 *Ercc1*$^{-/-}$ (P7 KO) MEFs. QRTPCR expression values are relative to WT Passage 3 (P3 WT) samples, which were normalized to a value of 1. **(B)** QRT-PCR analysis was performed on livers of WT young (20 weeks), progeroid *Ercc1*$^{-/\Delta}$ mice (20 weeks), and WT old mice (30 months) to examine Dicer mRNA expression. Dicer mRNA is downregulated significantly in WT Old mouse livers compared to WT Young and *Ercc1*$^{-/\Delta}$ livers. The mean of three independent mouse livers for each condition is graphed as fold-change expression relative to WT young livers, which were normalized to a value of -1. All experiments were performed in triplicate and the standard deviation is plotted as error bars. *P*-values were calculated using Welch's t-tests and are indicated by * (p < .05) and # (p< .0001).
